# Development and preliminary findings of a scoping review on the use of real‐world data in health services research for Alzheimer’s disease and related dementias

**DOI:** 10.1002/alz70861_108648

**Published:** 2025-12-23

**Authors:** Ashley Kuzmik, Arpita Tripathi, Laura M Block, Oluwagbemiga Oyinlola, Pedro JDMR Pinho, Zahra Rahemi, Pallavi Tyagi, Lycia Tramujas Vasconcellos Neumann

**Affiliations:** ^1^ Alzheimer's Association, Chicago, IL USA; ^2^ University of Wisconsin‐Madison, Madison, WI USA; ^3^ McGill University, Montreal, QC Canada; ^4^ Universidade Federal de São Paulo (UNIFESP), São Paulo, São Paulo/SP Brazil; ^5^ Clemson University, Greenville, SC USA; ^6^ University of Maryland, College Park, MD USA

## Abstract

**Background:**

The Alzheimer’s Association has prioritized advancing health services research (HSR) to improve care and outcomes for people living with Alzheimer’s disease and related dementias (AD/ADRD). HSR plays a critical role in investigating how care and treatment for AD/ADRD are accessed, delivered, and experienced. Real‐world data (RWD), including electronic health records (EHRs), claims, administrative, and registry data, offer opportunities to assess healthcare utilization, quality, and population‐level outcomes. This scoping review aims to map the use of RWD in AD/ADRD‐focused HSR in the United States and identify gaps and priorities to advance HSR and inform high‐value care. This review addresses the research question: *How is RWD being used in recent HSR focused on AD/ADRD in the United States?*

**Method:**

This review follows the Arksey and O’Malley framework and PRISMA‐ScR guidelines. A protocol was developed with input from experts across healthcare delivery, policy, public health, and psychosocial research, shaping the research question, database selection, keywords, and coding framework. A systematic search (2020–present) was conducted across PubMed, Embase, CINAHL, Web of Science, and EconLit. Seven reviewers independently screened articles using Covidence. Thematic analysis will be conducted using MAXQDA, followed by a consensus‐building workshop with healthcare ecosystem representatives to refine findings and inform future research.

**Result:**

A total of 2,354 articles were identified; 599 full‐text studies were assessed for eligibility, and 520 were included. Data extraction is ongoing. Preliminary findings show variation in RWD (e.g., claims, EHRs, registries), study designs, and populations. Common topics include healthcare utilization, treatment outcomes, and health and social characteristics of people with AD/ADRD. Studies span from diagnosis to end‐of‐life care. Some studies used quasi‐experimental or machine‐learning methods and novel sources, such as clinical notes, to examine outcomes. Gaps include limited data integration, underrepresented populations, and inconsistent evaluation of care models.

**Conclusion:**

This review provides an overview of how RWD is used in AD/ADRD‐focused HSR and highlights where future work is needed. Findings will support stronger use of RWD in scientific studies, improve treatment and support for people living with dementia, and inform healthcare planning and decision‐making across settings and populations.

